# Influence of pH on Soil Charge Characteristics and Cadmium Sorption in Some Noncontaminated Soils of Indian Subtropics

**DOI:** 10.1100/tsw.2005.26

**Published:** 2005-03-18

**Authors:** Tanmoy Karak, Dilip Kumar Das, Uttam Kumar Singh, Debtanu Maiti

**Affiliations:** ^1^ Department of Soil and Water Sciences, College of Resources and Environmental Sciences, China Agricultural University, Beijing-100094, P.R. China; ^2^ Department of Agricultural Chemistry and Soil Science, Faculty of Agriculture, Bidhan Chandra Krishi Viswavidyalaya, Mohanpur, Nadia-741252, West Bengal, India

**Keywords:** point of zero salt effect (PZSE), pH, Cd, sorption isotherm

## Abstract

Concentrations of total dissolved cadmium (Cd) and activity of its free ions in soil solution are suggested to be influenced by soil pH, organic matter (OM) content, cation exchange capacity (CEC), and clay mineralogy. We investigated the sorption of Cd by taking 25-, 50-, and 100-µM Cd solutions in five noncontaminated soils of West Bengal, India, having differing chemical properties with batch sorption experiments. The charge characteristics and point of zero salt effect (PZSE) of all experimental soils were calculated by the potentiometric titration method measuring the adsorption of H^+^ and OH^–^ on amphoteric surfaces in solutions of varying ionic strength (I). Sorption of Cd was more pronounced at pH levels greater than PZSE for all experimental soils. The CEC, OM content, clay mineralogy, and specific surface area (SSA) also had a great influence on the sorption of Cd from soil solution to soils. The relationships of Cd with those parameters were found to be consistent and the results concluded that Cd sorption in soils is strongly affected by the soil characteristics.

### INTRODUCTION

The increasing exploitation of the Earth's raw materials, coupled with the exponential growth of the world's population over the past several years, has resulted in environmental pollution, of which heavy metals are of great concern[1]. Soils are an important sink for these metals due to the high metal retention capacity of soil. Heavy metals, particularly cadmium (Cd), pose threats to soil quality and human health. People exposed to even low levels of Cd over a long period of time may develop kidney damage as well as damage to the lung, bone, cardiovascular system, liver, and reproductive system[2]. Cadmium enters the environment as a result of both natural and anthropogenic activities[3]. The natural process of pedogenesis leads to mineral breakdown and translocation of the products, as well as accessions from dust storms, volcanic eruptions, and forest fires. Often, the concentrations of Cd released into the soil system by pedogenic processes are largely related to the origin and nature of the parent materials. Anthropogenic sources comprise contributions made by mankind including industrial processes that contribute to both atmospheric and land depositions, mining and metallurgy, urban and industrial wastage, and sewage and fertilizer applications[4]. Soil characteristics positively correlated with Cd retention are pH[5], organic matter (OM) content, cation exchange capacity (CEC)[6], and specific surface area (SSA)[7], while free iron oxides are negatively correlated[8].

Knowledge of the charge characteristics of soils has high implications for the ion exchange, adsorption, and other chemical processes occurring at the liquid/colloid interface. Such reactions control various physicochemical properties of ions, particularly of Cd, and hence affect soil-water-plant ecosystems as well as the environment. For metal oxides and carbonates, the exposed surface determines the charge density. For these minerals, the ion exchange equilibrium at the solid-liquid interface depends on the strength of the associate coulombic forces. Soil minerals exhibit two types of charge: permanent or constant and variable or pH dependent. Constant charge is invariant with soil pH and results from isomorphous substitution and variable charge is due to protonation of the SiOH groups on the clay surfaces[9]. The variable charge component in soils changes with pH due to protonation and deprotonation of functional groups on inorganic soil minerals such as kaolinite, amorphous materials, metal oxides, oxyhydroxides, and hydroxides, and layer silicates coated with metal oxides and soil organic matter.

The soil is a heterogeneous mixture of different organisms and minerals where several factors control the mobility and availability of trace metals stemmed from industrial and natural resources. However, the magnitude of the charge generated in soil particles is the function of the SSA because this is the potential number of reactive groups exposed to the solution[3]. Most of the soil minerals bear two types of charges: constant and variable or pH dependent with relatively high surface areas ranging form 5–40 for kaolinite, 60–200 for soil goethites, to over 1000 $m^2g^{-1}$ for allophone. Surface charge density of a soil particle is also a function of SSA and diffused cloud of ions together with the charged surface which constitute an electrical double layer.

Unlike soils with permanent charge where the charge arises as a result of ismorphous substitution within the layer lattice of clay mineral, variable charge soils exhibit the property of being able to alter charge in response to changes in the ambient soil solution. Besides other factors that determine this variable charge, the major factors are pH and ionic strength (I)[3]. The variable charge in soils usually arises on the edges of lattice clay mineral and on the surfaces of sesquioxides, amorphous materials such as allophone, imogolite, and organic matter. The reactive groups responsible for variable charge are similar in all the inorganic colloids and different in OM (Fig. 1).

Most studies involving Cd have been limited to soils of temperate regions where the greatest proportion of soil is dominated by permanently charged surfaces with net negative charge. Soils have the capacity to store metal ions[10]. In contrast, strongly weathered tropical soils contain variable charge minerals that have low negative surface charge densities at field pH values (4.5–5.0). Such soils exhibit low affinities for heavy metals, especially at pH values close to the point of zero charge (PZC). The pH where the net total particle charge is zero is called the point of zero charge (PZC), which is one of the most important parameters used to describe variable-charge surfaces[11] and retention of metal on the soil surface. If the pH of a soil is above its PZC, the soil surface will have a net negative charge and predominantly exhibit an ability to exchange cations (CEC, exchange of one positive ion by another), while the soil will mainly retain anions (electrostatically) if its pH is below its PZC (AEC, exchange of one negative ion for another). pH may also correlate strongly with soil components such as clay minerals, oxides, or OM and hence musk their contribution to the Cd sorption[12]. Anderson and Christensen[13] found that pH was the most influential factor in controlling the distribution of Cd in soils. Almas et al.[14] reported that Cd sorption increased with pH, even within a very narrow range of pH values ($\Delta pH \approx 0.3$) in sandy and clayey soils of northern Germany. Although soil solution composition and concentration have strong effects on the sorption behavior of heavy metal in topsoils, and especially of Cd, pH is by far the important state variable in that regard.

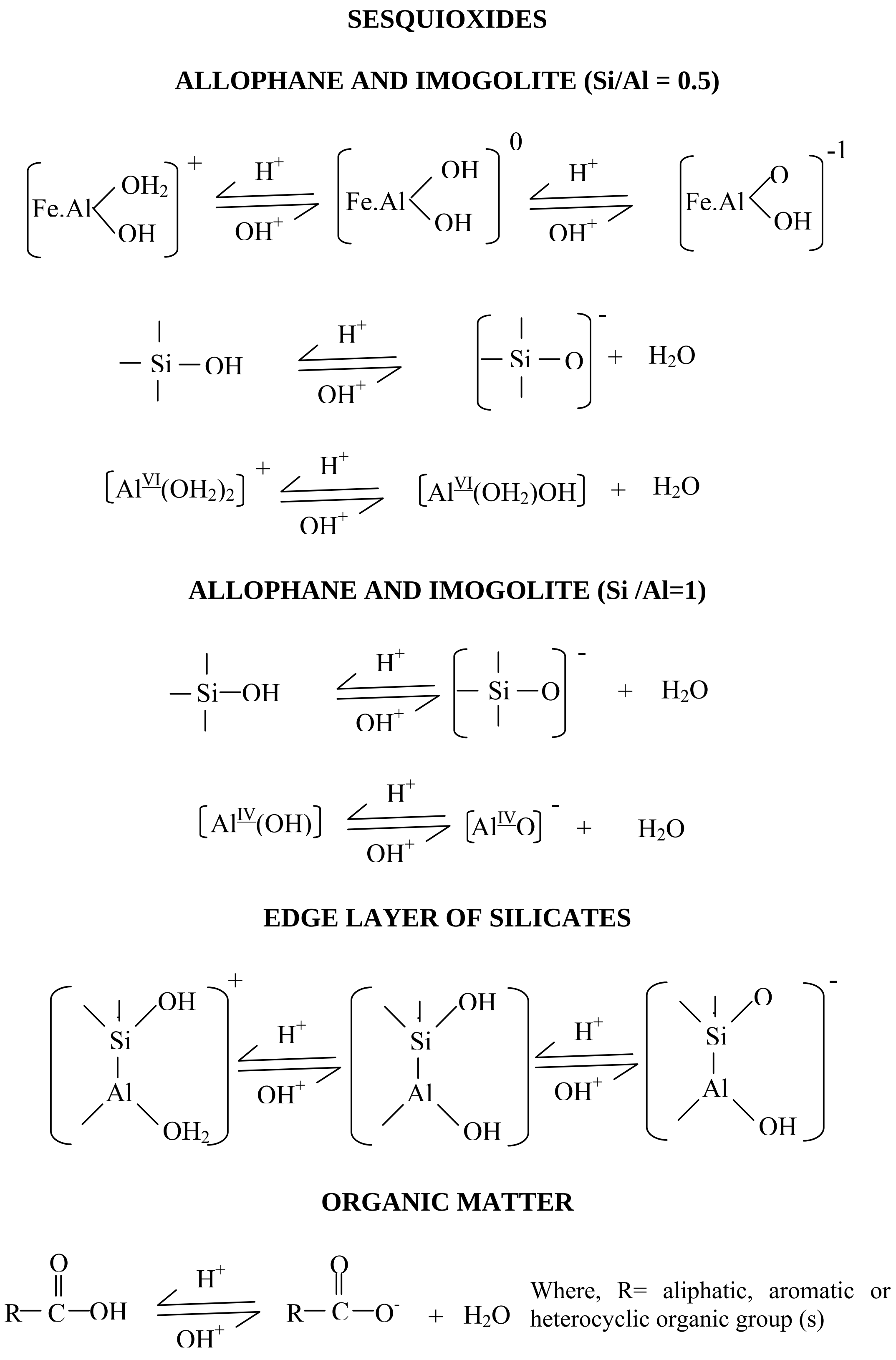

**FIGURE 1.** Effect of pH on charge generation in various inorganic colloids and organic matter. (In this study.)

Therefore, in order to understand the predictive capability of Cd retention in soils, the objective of this study was to relate the Cd sorption with several chemical and mineralogical soil characteristics for selected Indian soils.

## EXPERIMENTAL METHODS

### Soils and Soil Characterization

Some of the chemical and physical characteristics of the soil samples are listed in Table 1. The soils used in this study were collected from the surface horizon (0–15 cm) of five different zones of a subtropical region, namely Chondrokona (sample ID: CHA), Belda (sample ID: BEA), Jharul (sample ID: JHL), Diamond Harbour (sample ID: DIR), and Lakhsmikantapur (sample ID: LAR). The first three soil samples were from the western part of West Midnapore district of West Bengal and the last two were from South 24-praganas district of West Bengal. The soils were air dried and passed through a 2-mm sieve before use. The pH ($H_2O$) was measured potentiometrically in a 1:10 (soil:solution ratio in order to maintain the same ratio as that of the ion adsorption measurement) using a combination of glass-AgCl electrode. Exchangeable acidity was measured by using neutral 0.1*N* KCl solution maintaining 1:10 soil:solution ratio. Organic carbon content was determined by $K_2Cr_2O_7$ at digestion[15]. CEC of the soil sample was determined by the $NH_4OAc$ exchange method[16]. The particle size distribution was determined by sieving and pipette method after removal of OM with 30% $H_2O_2$ and dispersion in 0.002*M* sodium pyrophosphate[17]. SSA of soil samples was measured by a three-point Brunauer-Emmett-Teller (BET) isotherm for $N_2$ adsorption at –196°C on a quantasord sortometer (Quantachrom, Syosset, NY) using the following equation[18]:

$$\frac{\frac{p}{p_0}}{q(1-\frac{p}{p_0})} = \frac{1}{x_m C} + \frac{(C-1)}{x_m C}\frac{p}{p_0}$$

where $p$ is the pressure of the adsorbate, $p_0$ is its pressure at saturation (i.e., in equilibrium with bulk liquid phase of the adsorptive), $C$ is an empirical constant or conversion constant related to the enthalpy of the gas adsorption, $x_m$ is kg of adsorbate per kg of sample at monolayer coverage (i.e., monolayer adsorption capacity), and $q$ is the amount of gas adsorbed per mass sample. A semi-quantitative mineralogical analysis of experimental soil samples was carried out using a Philips X-ray diffractometer and Fe-filtered CoK$\alpha$ radiation at a scanning speed of $2^o 2\theta$ min$^{-1}$. The samples were rendered Ca-saturated, Ca-glucolated, K-saturated, and heated to 300 and 550°C. For the semi-quantitative estimation of clay minerals, the methodology proposed by Gjems[19] was adopted. Pyrophosphate extractable Fe and Al determination was performed according to the methods described by McKeague[20]; the acid ammonium oxalate extractable Fe, Al, and Si determined as described by Black et al.[21]; and the citrate-bicarbonate-dithionite (CBD)-extractable Fe, Al, and Si determined according to the method described by Mehra and Jackson[22]. Extractable Cd was determined by shaking 10 g of soil with 20 ml of solution containing 5 m*M* diethylenetriaminepentaacetic acid (DTPA), 0.01*M* $CaCl_2$, and 0.1*M* triethanolamine (TEA) adjusted to pH 7.3[23] for 2 h. The filtrates were analyzed for Cd using AAS.

**TABLE 1**
**Physicochemical Properties of the Experimental Soils**

| Parameters | | Location of Soils* | | | | |
|---|---|---|---|---|---|---|
| | | CHA | BEA | JHL | DIR | LAR |
| Taxonomic classification | | Typic Haplaquest | Typic Haplumbrept | Typic Ustochrept | Vertic Ustochrept | Typic Paleustalf |
| pH ($H_2O$); 1:2.5 | | 5.23 | 5.61 | 6.12 | 8.21 | 7.73 |
| pH (KCl); 1:2.5 | | 5.26 | 5.19 | 6.01 | 8.06 | 7.61 |
| EC ($dSm^{-1}$) | | 0.09 | 0.12 | 0.05 | 2.81 | 3.90 |
| Organic C (g $kg^{-1}$) | | 4.7 | 8.2 | 9.86 | 12.3 | 16.2 |
| Clay content (%) | | 6.12 | 8.9 | 12.2 | 22.7 | 30.9 |
| Textural classes | | Sand | Loam sand | Sandy loam | Sandy clay loam | Clay loam |
| Clay mineralogy (%) | Montmorillonite | 10 | 13 | 20 | 51 | 60 |
| | Vermiculite | 25 | 30 | 32 | 39 | 40 |
| | Kaolinite | 55 | 45 | 20 | 10 | - |
| | Biotite | 10 | 12 | 28 | - | - |
| CEC ($cmol^+$ $kg^{-1}$) | | 1.5 | 2.8 | 6.2 | 16.1 | 18.7 |
| Surface area ($m^2g^{-1}$) | | 40.4 | 42.2 | 45.4 | 49.8 | 51.6 |
| Pyrophosphate extracted Fe (mmol $kg^{-1}$) | | 42.1 | 39.4 | 38.1 | 2.3 | 1.1 |
| Pyrophosphate extracted Al (mmol $kg^{-1}$) | | 91.2 | 62.3 | 45.6 | 0.9 | 0.2 |
| Water soluble $CO_3^{-2}$ (g $kg^{-1}$) | | 0.01 | 0.03 | 0.41 | 15.1 | 9.8 |
| DTPA extractable Cd (mg $kg^{-1}$) | | ND† | 0.03 | 0.02 | 0.05 | ND |

* See text for explanation of sample ID abbreviations.

† ND, Not detectable.

### Charge Characterization

The methodology of van Raij and Peech[24] was used. Soil samples weighing 1.5 g were suspended in 15 ml of 0.1, 0.01, and 0.001*M* KCl, and pH adjusted to the desired value by the addition of a measured amount of HCl or KOH. pH ranges adjusted for charge characterization on experimental soils were from 2.1–9.5 for CHA, 2.3–9.7 for BEA, 2.2–11.5 for JHL, 4.4–11.2 for DIR, and 4.2–11.4 for LAR. The suspensions were kept at room temperature (24 ± 2°C) in capped 20-ml plastic vials and shaken twice daily over a 3-day period. After this period, the pH values of the supernatant were recorded. Subsequently, the samples were washed three times with 25-ml KCl solution selected for saturation and adjusted to the desired pH, followed by five more washings with 25 ml of the same salt solution. Some readjustment of the pH was necessary with HCl or KOH before the last one or two washings. After the final centrifugation, the centrifuge tube containing the washed soil was drained and, in order to determine the occluded solution, volume was immediately weighed. The pH of the final extract was determined and considered to be the equilibrium pH. The sample was then extracted five times with 25 ml of 0.5*M* $Na_2SO_4$ of KCl-saturated samples. Potassium was determined by flame photometry and chloride ($Cl^-$) was determined by titration with $AgNO_3$ in the presence of potassium chromate ($K_2CrO_4$) as an indicator of the end point of titration[17]. The amounts of $Cl^-$ and $K^+$ extracted, corrected for the occluded KCl solution, were expressed as equivalent amounts of positive and negative charges, respectively.

### Sorption of Cd as a Function of pH

Sorption of Cd was determined at varying pH values (which were adjusted for charge characterizations) including respective PZSE of experimental soils to elucidate pH effects on surface charge and Cd sorption in the soils. Soil solution containing approximately 1.5 g of soil and 29 ml of 0.007*M* KCl were prepared in 50-ml polyethylene centrifuged tubes. Suspension pH values were attained by adjustment with either HCl or KOH. The solutions were shaken for 24 h at 25 ± 2°C, after which the pH was measured and readjusted if necessary. This was performed until the suspension pH values were stable at the desired levels; 1 ml of Cd as $CdCl_2$ was added with varying concentrations of Cd. The final Cd concentrations used were 25, 50, and 100 μ*M*. After Cd addition, the solution was shaken on a temperature control, reciprocating shaker for 24 h at 25 ± 2°C to reach the equilibrium; 24 h were sufficient to achieve the equilibrium and that time was confirmed from the preliminary kinetic studies. Suspension pH was measured and the samples were centrifuged. The supernatant was collected and analyzed for Cd under AAS. Blank solutions containing only 100 μ*M* $CdCl_2$ in 0.01*M* KCl was titrated with KOH to check for formation of solid phases. The addition of KOH yielded no precipitate in the presence of Cd within the experimental pH range of this study and, therefore, losses of Cd from soil solution to soil were considered to be a sorption characteristic in soils. The soil solid-phase Cd was calculated by subtracting the Cd; those in solutions after equilibration from initial Cd concentrations.

### Evaluation of Sorption Data

Vertical displacement and leaching of heavy metal as well as their root uptakes in soils are governed by their interaction between a multiplicity of sorption site and the soil solution. The interaction is expressed by sorption isotherms acting as “adjusting parameters functions”. The relation between desorbed and sorbed heavy metal concentrations may be expressed by the Freundlich isotherm.

$$S = kC^M$$

where $S$ is the heavy metal concentration in the sorbed phase (μ*M*/kg soil), $C$ is the heavy metal concentration in the solution phase (μ*M*/l), and $k$ ($L^M \mu M^{1-M}$/kg soil) and $M$ ($0 < M < 1$) are parameters. Although soil solution composition and concentrations have strong effects on the sorption behavior of heavy metal in topsoils and especially of Cd, pH is by far the most important state variable in that regard, followed by OM content proposed by van der Zee and van Riemsdijk[25]

## RESULTS AND DISCUSSION

### Effect of pH and KCl Concentration on Charge Distribution on Soil Surface

The charge distribution for the various soils studies, as determined by measuring the adsorption of $K^+$ and $Cl^-$ from KCl as a function of pH, are presented in Fig. 2. The curves at different ionic strengths intersect at a common point that is the PZSE; at this pH, the net charge is independent of ionic strength when pH is plotted against surface charge or amounts of acid or base added. The five soils clearly demonstrated points where salt concentration had no effect on adsorption of potential determining ions (i.e., $H^+$ and $OH^-$) over the pH ranges measured (Fig. 2). The positive charge decreases and negative charge increases with increase in pH for all experimental soils. The maximum positive charge developed at low pH values for all soils. PZSE values were found 4.2, 5.4, 6.3, 7.8, and 8.5 for the soils CHA, BEA, JHL, DIR, and LAR, respectively. For all acidic soils (CHA, BEA, and JHL), the PZSEs were found to be in the acidic range and for others (DIR and LAR) in the basic range. Lower PZSE values in acidic soils reflect the

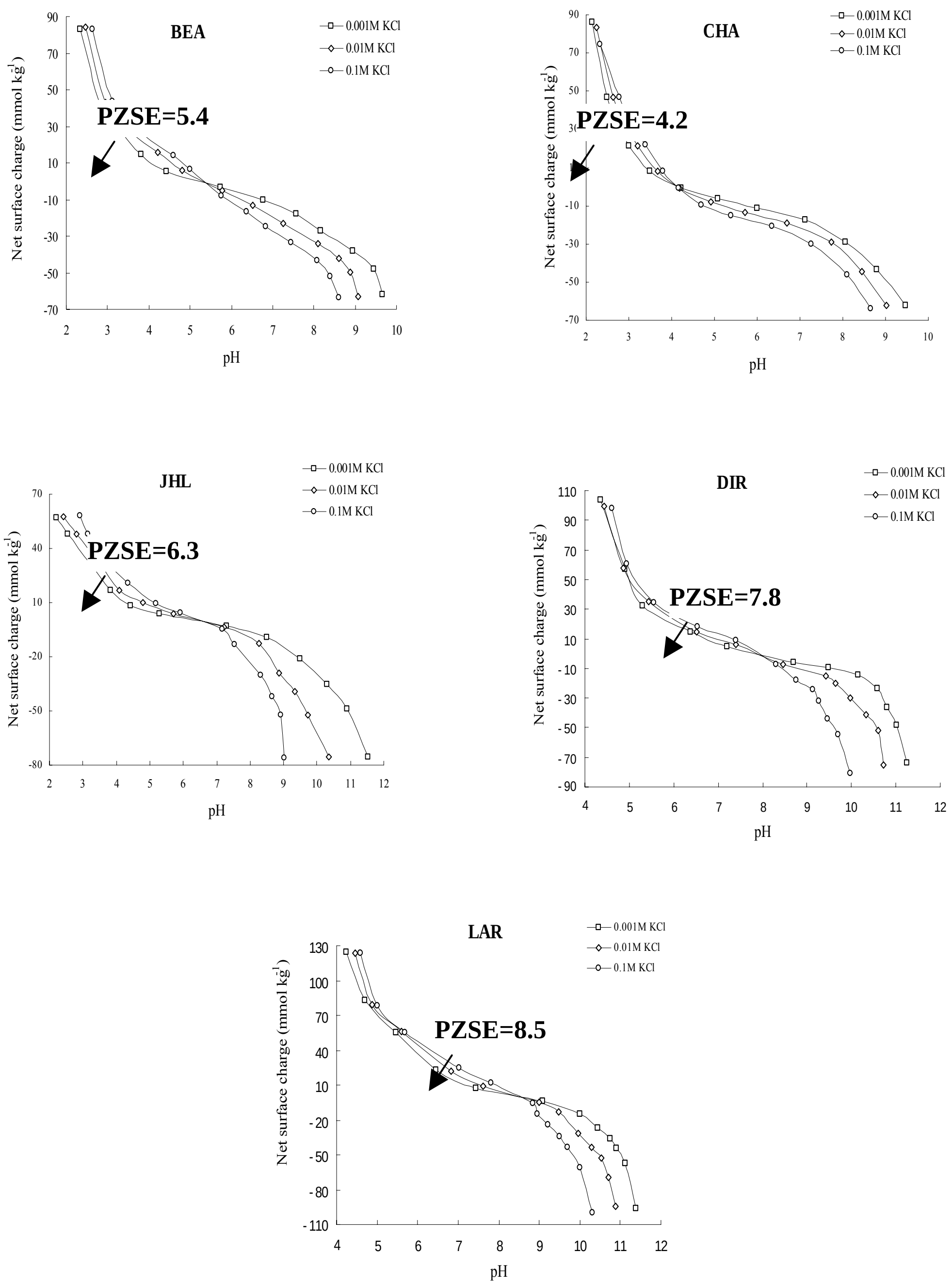

**FIGURE 2.** Determination of surface charge in experimental soils by potentiometric titration at three different concentrations of KCl.

effect of higher OM[26], whereas higher PZSE values in alkaline soils reflect the effect of lower OM in the soil. The curves of the soil samples did not follow the usual trend of higher electrolyte concentration resulting in higher pH below the PZSE because at lower pH values, protons were primarily consumed in the mineral dissolution reactions present in soils instead of generating surface charge. This would have the effect of narrowing the margin of difference between various electrolyte concentration curves, which was evident. It is postulated that the effect described above was not as important for the soils BEA and JHL, which had different mineralogy (Table 1). Moreover, all the samples demonstrated increased separation between curves at pH values above the PZSE. Above this point, material buffering capacity is reduced, which is manifested via relatively small increases in pH, resulting in large expansion in charge generation[27]. The lower PZSE values of acidic soils are due to some permanent surface-charge minerals, which gives rise to a slightly lower PZSE[11]. The pH effect in the positive and negative charges of soil is probably due to proton transfer mechanism on OM, Fe and Al oxides, and edges of kaolinite[28]. The negative charge at low pH values is the result of a permanent negative charge arising from isomorphous substitution within the lattice of clay crystal. Evidence for isomorphous replacement within the lattice clay mineral framework of kaolinite has actually been demonstrated by Robertson et al.[29]. Differences in amounts of cation and anions absorbed in soils and between horizons of the same soils at a given pH must be explained through differences in the quantities and characteristics of their exchange materials. As the electrolyte concentrations decrease, the magnitude of positive, negative, and net charges also decreases. The effect is more pronounced in soils BEA and JHL and it usually occurs at the extremes in the pH range; i.e., very high or very low pH. For the other soils (CHA, DIR, and LAR), the electric charges were more or less the same at both concentrations of KCl on the lower of their PZSE. This is especially true for soils DIR. In all instances, the net negative charges increase with pH whereas the net positive charges decrease. The concentration effect on the electric charges is of less importance than the pH effect. Differences at high pH may be related to interactions between exchangeable Al, OM, and possible mineral matter. At the lower pH, the differences may be due to some permanent charge occupied by hydroxyl Al ions that are released at the higher concentration of KCl, but not at the lower ones. Nye et al.[30] reported that K is selectively bonded over Al when the concentration of KCl was 1*N*. The reverse was true with more dilute KCl solution. The charge at a particular pH is obtained as the difference between positive and negative charges. The net charge is more strongly pH dependent because a variable component of positive charge has been superimposed on the stronger pH-dependent negative charge[28].

### Effect of pH on Cd Sorption in the Soil

The pH-dependent Cd adsorption behavior of the five soils is shown in Fig. 3. Cd adsorption isotherms of five different topsoils cover the pH range from 2.14–9.46 for CHA, 2.34–9.66 for BEA, 2.20–11.52 for JHL, 4.36–11.25 for DIR, and 4.24–11.39 for LAR. Adjusted pHs were included in the PZSE of all the experimental soils. The similar trends of Cd adsorption were found for all experimental soils. Although the extent of adsorption varied among all pH ranges, a common pH-dependent trend was frequently observed for sorption of Cd from dilute aqueous solutions. Cadmium sorptions for all experimental soils were lower in acidic range pH. However, Cd sorptions were shifted drastically to higher sorption even within the very narrow range of pH values after reach the PZSE. In general, Cd adsorption for all experimental soils was found more pronounced on the pH range greater than the soil's PZSE. The 50% of preloaded Cd was adsorbed in soil at higher adjusted pH for all soils. The nature of sorption isotherm (pH < PZSE) clearly follows the linearity of Langmuir sorption isotherm, but the nonlinear sorption curve. The nature of sorption curves was similar to those found in the Adhikari and Singh[31] study on the sorption characteristics of Cd in some selected soils of India. The effect of pH on Cd adsorption in soils is manifested through changes in surface charge density of the variable charge minerals. Increasing soil solution pH leads to a rapid increase in net negative surface charge (Fig. 2) that may explain the accelerated affinity for Cd ions[3]. Cadmium sorption was greater in soil LAR (PZSE = 8.5). These results clearly show that the soil suspension pH is considered to be one of the most dominating factors controlling the Cd sorption in soils, which is also consistent with the studies of Garcia-Miragaya and Page[10]. Sharp variations of Cd sorption at the pH greater than PZSE have commonly been attributed to the preferential adsorption of $[CdOH]^+$[32], the adsorption of $Cd^{+2}$[33], proton competition on adsorption site[34], and type of clay mineralogy[35]. Increasing solution pH leads to a rapid increase in net negative surface charge that may explain the enhanced affinity for metal ions. The amount of Cd adsorption was found greater at pH values above the PZSE point (Fig. 2). In all experimental soils, the sorption of Cd was lower in pH of PZSE.

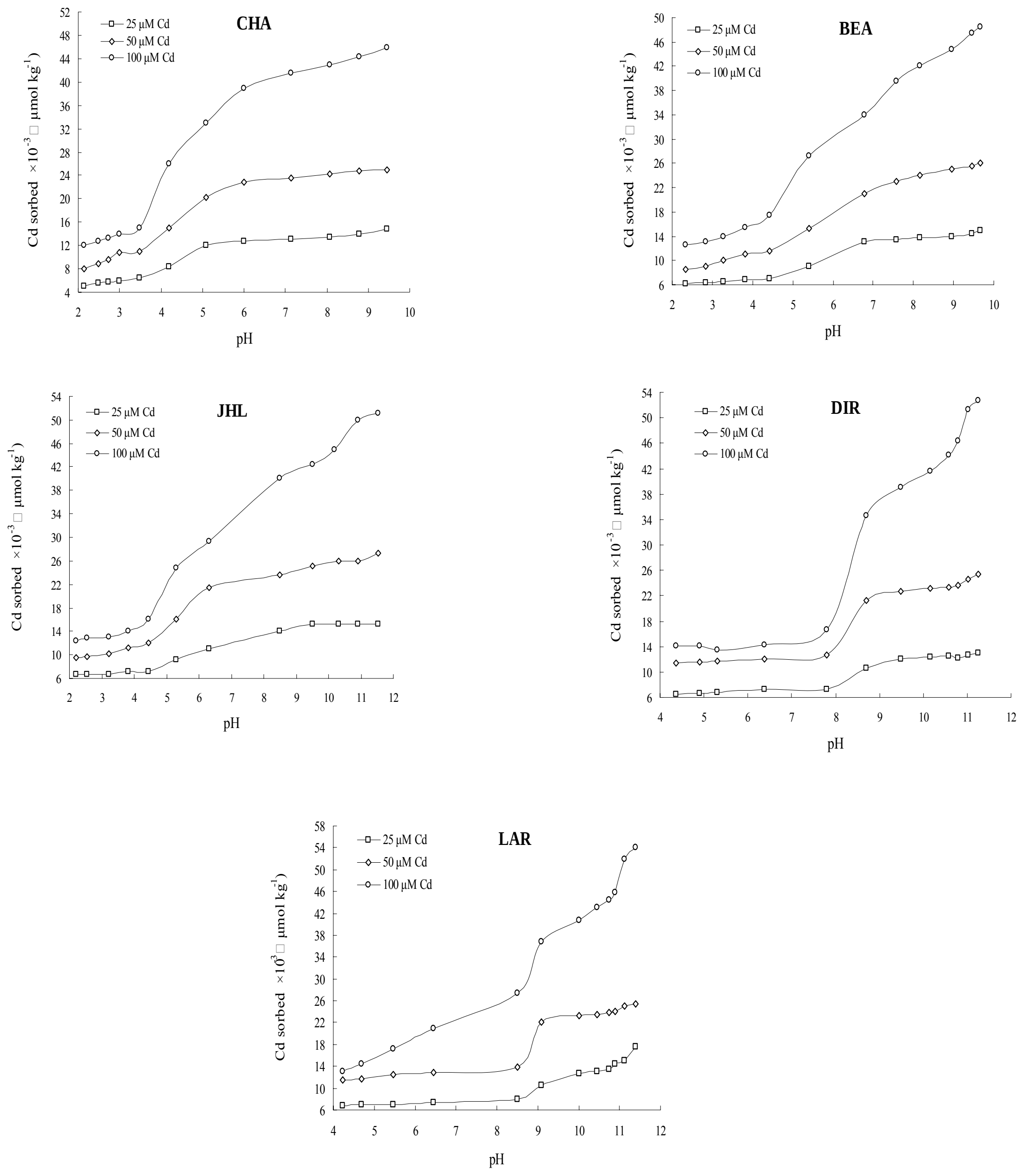

**FIGURE 3**. Effect of pH on Cd sorption in soils.

This is an expected result because at the higher pH, all experimental soils exhibit negative charge that influences the electrostatic interaction between soil solution $Cd^{+2}$ ions and charge clay particles. Although pH plays a vital controlling factor in Cd sorption, it may also correlate strongly with soil components such as clay minerals and OM and hence mask their contribution to the Cd sorption[12]. Due to various component interaction and competition for Cd sorption, it is unclear which components govern Cd sorption in whole soils.

In this experiment, it was found that sorption of Cd was increased with increase of soil CEC. The increasing order of CEC were LAR (18.7) > DIR (16.1) > JHL (6.2) > BEA (2.8) > CHA (1.5) (Table 1). This is an expected result because Cd adsorption takes place by an ion exchange process in clay minerals[35].The results were in agreement with the studies of Zachara et al.[36]. The expected reasons are that adsorption of Cd on hydroxylated sites on the edges of the layer silicates or on Fe or Al oxides were controlled by the CEC at acidic pH range. The finding is also consistent with the SHAB (soft hard acid base) theory in which the reactive surface sites of Fe oxides are hard in character ($r_{Fe+3}$ = 0.073 nm and $r_{Fe+2}$ =0.078 nm) whereas Cd ($r_{Cd+2}$ = 0.095 nm) is a soft acid and is not expected to react strongly with the oxide surfaces in acid soils, i.e., CHA, BEA, and JHL[37]. On the similar fashion, $r_{Al+3}$ = 0.053 nm, which does not permit the stable interaction with soft acid like Cd. Due to that fact, the lower sorption of Cd was found in acidic soils (CHA, BEA, and JHL) rather than that of an alkaline one (LKR and DIR**).**

From the mineralogical viewpoint, the soil with a higher amount of kaolinite shows the least amount of Cd sorption due to minimal surface area (7–30 $m^2$/g ) and CEC (3–22 cmol(+)/kg), which are in agreement on the study of Ziper et al.[35,38]. Kaolinite has only edge and planer surfaces, of which only edge sites contribute the CEC**.** Biotite is a better Cd adsorbent than kaolinite because both the edge and planer surfaces contribute to CEC. The contribution from interlayer is extremely small because of high selectivity of $K^+$ over $Cd^{+2}$[35] and can be ignored. Montmorillonite and vermiculite have interlayer exchanges sites in addition to the edge and planar sites and that is why these minerals adsorbed the most Cd. Thus, the results with constant external surface area also reflect the total exchange capacity of the mineral. Kabata-Pendias[38] showed that montmorillonite and vermiculite exhibited surface area and CEC as 280–800 and 50–800 $m^2$/g and 80–150 and 10–200 cmol(+)/kg, respectively. Surface measured by $N_2$ adsorption does not count interlayer surface making vermiculite and montmorillonite appear to be stronger adsorbents than kaolinite and biotite for Cd. Montmorillonite shows the least amount of $Cd^{+2}$ sorption on a total surface area basis.

### CONCLUSIONS

The study shows that soil chemical characteristics can have a marked effect on the adsorption of Cd in soils. The present study confirms that an empirical relation may be used successfully in discerning the relative role of $H^+$ concentration on Cd adsorption. The effect of soil solution composition on Cd sorption in soils is an important factor and must be considered during the assessment of Cd behavior. The findings may have major implications towards Cd sorption in soils that have inherently low affinity for Cd. Such soils include sandy or highly weathered soils, which despite their high clay contents, may have high Cd exchange capacities due to their surface chemical properties. In such soils, an adequate consideration of solution composition effects on Cd sorption is imperative not only from the point of view of the residence time of Cd in the root zone (thereby its accumulation or plant uptake), but also for its potential to contaminate groundwater.

### BIOSKETCHES

**Tanmoy Karak** has published about twelve research papers in the fields of soil chemistry and plant nutrition. Dr. Karak has been nominated as a visiting Soil Scientist for the Indo-China academic exchange program during the period 2003–2005.

**Dilip Kumar Das**, Professor in the Department of Agricultural Chemistry and Soil Science, has about 25 years of experience in teaching in the undergraduate and postgraduate levels, and research relating to soil chemistry, soil fertility, and plant nutrition with particular reference to micronutrients including heavy metals. He has also guided a good number of Ph.D. and postgraduate students for the award of their degrees. Prof. Das has published about 100 original research papers in national and international journals of repute and has also published seven books from Kalyani Publishers, India. http://myprofile.cos.com/dkdas

**Uttam Kumar Singh** has published about twelve original research papers in journals of national and international repute. Dr. Singh has been nominated as a visiting Soil Scientist for the Indo-China academic exchange program during the period 2003–2005.

**Debtanu Maiti** is a Ph.D. student whose fields of specialization are soil chemistry, soil fertility, and plant nutrition. Sri Maiti has published about eleven original research papers in journals of national and international repute.
